# The neurobiology of attachment and the influence of psychotherapy: a literature review

**DOI:** 10.1192/bjo.2021.759

**Published:** 2021-06-18

**Authors:** Graziella Romano, Daniela Patrascu, Priyanka Tharian, William Burbridge-James

**Affiliations:** 1 ST6 General Adult Psychiatry and Medical Psychotherapy, South London and Maudsley NHS Foundation Trust; 2 Consultant in General Adult Psychiatry and Perinatal Psychiatry, Essex Partnership University NHS Foundation Trust; 3 Consultant in General Adult Psychiatry, East London NHS Foundation Trust; 4 Consultant Psychiatrist and Medical Psychotherapist, Essex Partnership University NHS Foundation Trust

## Abstract

**Aims:**

To review the existing scientific literature on the neurobiology of caregiver-infant attachment and the effects of psychotherapy on neurobiological structures. We hypothesised that the therapeutic relationship is a new attachment relationship that can model and re-map neural networks involved in emotional self-regulation.

Understanding attachment is relevant to working with women and families in the perinatal period and has an impact on treatment outcomes. Evolutionary perspectives show that the infant's attachment to the caregiver is important for survival, development of self and relational patterns. Mother's attachment predicts the infant caregiving behaviour in perinatal period and psychotherapeutic interventions at this time have a role in modifying the risk of intergenerational transmission of trauma and further pathological attachment styles.

**Method:**

We performed a MEDLINE search focussing on the past 10 years. Keywords used were attachment, neurobiology and psychotherapy. We included original studies and existing reviews looking at all types of formal psychotherapy used and focussing on human research. Exclusion criteria were non psychotherapeutic interventions and attachment based on couples only.

**Result:**

There has been an increasing focus in the literature on studying the neurobiology of attachment in caregivers and infants both in healthy cases and in psychopathology over the past decade. Existing studies concentrate on care givers, there is growing evidence on the effects of attachment styles on the infant's brain, mostly from animal studies. Some authors looked at the effects of parental childhood trauma on later parenting styles and intergenerational transmission of trauma. A few studies highlighted neurobiological changes as a result of psychotherapeutic interventions in various psychiatric disorders.

**Conclusion:**

There is growing evidence on the neurobiology of attachment focussing on specific neurotransmitters and brain pathways. The modulating effect of psychotherapy has also been studied, albeit with more focus on recovery from psychiatric illness. The literature on neurobiological changes with psychotherapy remains scarce and heterogeneous and further research may be needed in the neurobiology of therapeutic relationship itself as there is increasing recognition that this may be the agent of change, with evidence in the role of linking cortical structures to subcortical limbic systems.

